# Running Footwear and Impact Peak Differences in Recreational Runners

**DOI:** 10.3390/biology11060818

**Published:** 2022-05-26

**Authors:** Federico Roggio, Bruno Trovato, Marta Zanghì, Luca Petrigna, Gianluca Testa, Vito Pavone, Giuseppe Musumeci

**Affiliations:** 1 Human, Histology and Movement Science Section, Department of Biomedical and Biotechnological Sciences, University of Catania, Via S. Sofia n°87, 95123 Catania, Italy; federico.roggio@unict.it (F.R.); bruno.trovato@phd.unict.it (B.T.); marta.zanghi@phd.unict.it (M.Z.); luca.petrigna@unict.it (L.P.); 2 Sport and Exercise Sciences Research Unit, Department of Psychology, Educational Science and Human Movement, University of Palermo, Via Giovanni Pascoli 6, 90144 Palermo, Italy; 3 Section of Orthopaedics and Traumatology, Department of General Surgery and Medical Surgical Specialties, University Hospital Policlinico “Rodolico-San Marco”, University of Catania, 95123 Catania, Italy; gianluca.testa@unict.it (G.T.); vpavone@unict.it (V.P.); 4 Research Center on Motor Activities (CRAM), University of Catania, Via S. Sofia n°97, 95123 Catania, Italy

**Keywords:** running gait, foot strike pattern, 3D motion analysis, markerless, prevention, sports performance

## Abstract

**Simple Summary:**

Running is a physical activity practiced by many people to maintain good levels of movement. Recreational runners commonly strike the ground with the postero–lateral zone of the foot, which may be associated with a higher biomechanical load on the lower limb, called impact peak. Different running shoes with specific cushioning are available to overcome the biomechanical load, e.g., shoes with a thickness difference between the forefoot and heel parts of the sole, called heel-to-toe drop. Analyzing the running pattern of recreational runners may be challenging because biomechanics laboratories mainly analyze these characteristics in individuals with visible alterations. To overcome these limitations, we employed a 3D markerless system; furthermore, we investigated footwear use. These parameters were studied to understand the behavior of those runners with and without a higher impact peak. Thirty participants underwent a running analysis and a questionnaire about their footwear. The study’s main finding highlighted kinematic and spatiotemporal differences between the runners presenting a higher impact peak and those without it. Furthermore, we observed that runners without an impact peak prefer shoes with a lower heel-to-toe drop, while the other group prefers shoes with a higher heel-to-toe drop. Investigating biomechanics characteristics is essential to reduce possible injury.

**Abstract:**

Running is a physical activity and the investigation of its biomechanical aspects is crucial both to avoid injuries and enhance performance. Recreational runners may be liable to increased stress over the body, particularly to lower limb joints. This study investigates the different running patterns of recreational runners by analyzing characteristics of the footwear impact peak, spatiotemporal, and kinematic parameters among those that present with a peak impact and those that do not, with a 3D markerless system. Thirty recreational runners were divided into two groups: impact peak group (IP) (*n* = 16) and no impact peak group (*n* = 14) (n-IP). Kinematic and spatiotemporal parameters showed a large Cohen’s d effect size between the groups. The mean hip flexion was IP 40.40° versus n-IP 32.30° (d = −0.82). Hip extension was IP 30.20° versus n-IP 27.70° (d = −0.58), and ankle dorsiflexion was IP 20.80°, versus n-IP 13.37° (d = −1.17). Stride length was IP 117.90 cm versus n-IP 105.50 cm (d = −0.84). Steps per minute was IP group 170 spm, versus n-IP 163 spm (d = −0.51). The heel-to-toe drop was mainly 10–12 mm for the IP group and 4–6 mm for the n-IP group. Recreational runners whose hip extension is around 40°, ankle dorsiflexion around 20°, and initial foot contact around 14°, may be predisposed to the presence of an impact peak.

## 1. Introduction

Running is one of the most common ways to practice physical activity, and it is estimated that almost 50 million Europeans practice this sport to stay healthy [1]. The typical strike pattern among runners is the rearfoot, defined as a pattern where the runner contacts the ground with the lateral portion of the heel [2]. Xu et al. [3] found that a rearfoot strike pattern has a higher general biomechanical load on the knees, patellofemoral joint, and over the ground. Rearfoot runners deal with a repeated ground impact during the first 50 milliseconds of the stance phase, which is an abrupt collision around 1.5–3 times the bodyweight [4]. The magnitude of this high load impact travels all over the body and can contribute to the onset of running-related injuries. Modern running shoes can mitigate the perception of impact, but it may not disappear entirely [4].

Recreational runners may also encounter this due to potential inexperience. Furthermore, rearfoot runners appear to produce a higher magnitude and earlier vertical impact-peak timing than forefoot runners [4]. A meta-analysis [5] conducted among different categories of runners stated the incidence of running-related injuries with a weighted estimation of 7.7 (95% CI 6.9–8.7) per 1000 h of running in recreational runners. Different authors support the idea that alterations to running biomechanics may induce repetitive atypical load to the tendons that is associated with an increased risk of lower limb tendinopathies such as Achilles tendinopathy, iliotibial band syndrome, plantar fasciitis, and posterior tibial tendon dysfunction [6,7,8,9].

Different types of running shoes, i.e., minimalist or maximalist, have been produced to overcome the risk of running-related injuries [10], based on a difference in the thickness of the forefoot and heel parts of the sole, called heel-to-toe drop (HTD). The shoes with a low HTD have a measure of 4–6 mm, while the shoes with a high HTD can reach the 10–12 mm. A high impact peak is believed to be strictly related to running-related injuries [4], hence the need to produce shoes with increased cushioning, i.e., high HTD. However, this condition is still debated because there is no clear evidence that high cushioning can reduce the impact peak [11]. Furthermore, different HTD can induce different running biomechanics such as an increased vertical loading rate in low HTD [12], changes in the foot inclination angle and therefore, changes in the running biomechanics [13].

Evaluation of running biomechanics is often performed to estimate the characteristics of a running pattern to understand the relationship between kinematic variables that may predispose runners to experience injuries. In this context, the optoelectronic infrared multi-camera motion analysis system is the most accurate approach for analyzing movement [14]. However, these systems are expensive and subject to certain conditions such as a dedicated laboratory, long preparation times, or highly trained clinicians identifying the anatomical landmarks correctly to place the reflective markers [15,16]. To overcome some of these limitations, instrumented treadmills [17,18,19] and inertial measurement unit (IMU) systems [20,21] are spreading as a valid alternative to accomplish gait or run analyses in different environments, overcoming some of the previously mentioned limitations. In the context of recreational runners, this approach can be used to identify the alterations of the motion without excessive clinical effort. Knowing which biomechanical variables result in an altered running gait can guide runners or coaches toward an intervention to avoid the impact peak presence [22]. These new motion analysis technologies can provide a cost-effective and easily reproducible approach due to a 3D camera that detects kinematic variables and load cells that measure the vertical ground reaction force, used to derive spatiotemporal parameters.

This study aimed to collect runners’ characteristics related to their footwear and then employed an instrumented treadmill with a 3D camera to analyze the spatiotemporal and kinematics parameters of recreational runners. Furthermore, the runners were classified based on the presence or absence of an impact peak, the correlation among the measured parameters was then investigated.

## 2. Materials and Methods

This retrospective study involved thirty adult male half-marathon recreational runners (mean ± SD); age 46.28 ± 6.49 years, height 174.59 ± 5.87 cm, body mass 71.86 ± 6.77 kg, and BMI 23.56 ± 1.69 kg/m^2^. The running experience was 9.4 ± 2.2 years, and the km-average per week was 13 ± 3.4 km. Participants were recruited voluntarily at the Research Center in Motor Activities (CRAM), University of Catania. The exclusion criteria were recent joint trauma, pain during running, and history of professional running. Once we performed the running analysis, we classified the participants into two groups: impact peak presence (IP) (*n* = 16) and impact peak absence (n-IP) (*n* = 14). The data collection was approved by the Research Center in Motor Activities (CRAM), University of Catania (protocol n.: CRAM-09-2020, 16 March 2020), in accordance with the Declaration of Helsinki. All participants provided informed consent before participating.

### 2.1. Data Collection

A markerless system was used to analyze the kinematic movements in the sagittal plane. The Walker View (TecnoBody^®^, Dalmine, Italy) is a treadmill with a markerless system that automatically identifies anatomical landmarks through AI [15] valid for both spatiotemporal parameters and angular displacements [18,23,24]. It is composed of an instrumented treadmill equipped with eight load cells (composed by strain gauges, sampling frequency 100 Hz) and a 3D camera for motion capture (Microsoft Kinect v2, sampling frequency 30 Hz) available for sports, medicine, rehabilitation, and gait analysis. Eltoukhy et al. [24] reported excellent interclass correlation coefficients (>0.75) for agreement (ag) and consistency (cn) by comparing the measurements of this camera with a BTS optoelectronic system. Total hip ROM, ag = 0.80, cn = 0.86; total knee ROM, ag = 0.80, cn = 0.82; step length, ag = 0.67, cn = 0.87; contact time, ag = 0.82, cn = 0.97; CoM vertical displacement, ag = 0.83, cn = 0.83 [24]. Ankle dorsiflexion/plantarflexion, initial contact, and toe-off measurements were collected through two inertial measurement units (IMU) placed over the feet with a belt, connected via bluetooth to the system; weight 47 g, sampling frequency 100 Hz. For the running analysis, participants were advised to wear shorts, a t-shirt, and their own running shoes, leaving the anatomical landmarks uncovered. Before the test, they warmed up for 10 min on the treadmill at a self-paced speed according to their overground running speed. We used the adopted speed to set the run analysis later. The test was performed by keeping the erect position for a few seconds so that the system could locate the anatomical landmarks correctly. Once the exam was started, the runners had to run for 10 min, where the speed slowly increased until their preferred speed was reached. Then, the kinematics were recorded for 60 s. We divided the participants into the IP or n-IP groups based on the presence of the impact peak by visualizing the gait graph of the vertical load provided by the TecnoBody software.

Furthermore, runners completed a questionnaire to collect specific information related to their footwear and to investigate whether they had experienced injuries during the last year. We asked them information about the size of the shoes, how often they change the shoes, if they use any particular sole, if they experience pain after a training, kilometers per week, if they experienced injuries during the last year and if yes, then the injury location and severity. The complete questionnaire is presented as Supplementary File S1.

### 2.2. Data Processing

The integrated software (i.e., TecnoBody Management System, Bergamo, Italy) analyzes spatiotemporal and kinematic parameters. The system records each phase of the running cycle and then produces the report showing the averages of the joint ROM of the trunk, hip, knee, and ankle, and maximum extension and flexion values for each joint for both limbs. The quaternions of each anatomical part are calculated starting from the position of the articular joints. Then, they are decomposed into Euler angles following the International Society of Biomechanics guidelines for angle calculation [25]. Hereafter we refer to joint parameters as maximum extension and flexion. Spatiotemporal parameters included stride length, step time, step cycle, and vertical center of mass displacement (CoM), calculated with the segmental analysis method using lower body kinematic data and anthropometric measurements [26]. Furthermore, we calculated steps per minute (spm) as D/MSL/T, where D corresponds to distance traveled expressed in meters; MSL is the mean of stride length of the left and right feet expressed in meters; T is the total time of the run analysis, expressed in minutes.

### 2.3. Data Analysis

Statistical analysis was performed using R Project for Statistical Computing (Vienna, Austria). The data from the questionnaire were processed with descriptive analysis while the data of running biomechanics were processed through inferential analysis. The Shapiro–Wilk test verified the normality distribution; the Levene’s test verified the homogeneity of the variance; the Student *t*-test and the Mann–Whitney U test determined whether any significant differences in kinematics, spatiotemporal anthropometric, and demographic parameters existed between the groups. The Mann–Whitney U test was used as not all the variables were found to be normally distributed according to the Shapiro–Wilk test. Cohen’s effect size (d) was applied to identify meaningful differences between the groups. Based on Cohen’s criteria, d ≥ 0.80 (absolute value) was considered a large effect size, and d ≥ 0.50 (absolute value) was considered a medium effect size. Pearson correlation coefficients (r) between variables were calculated for each group separately to determine which kinematic and spatiotemporal parameters were related. A correlation matrix was arranged to present the existing correlations. Only significant correlations according to *p*-value < 0.05 were considered.

## 3. Results

Participant characteristics are shown in Table 1. No statistical differences were present among the two groups with regard to the anthropometric characteristics.

### 3.1. Spatiotemporal

Spatiotemporal results are reported in Table 2. The n-IP runners have a shorter stride length (105.50 ± 20.50 cm) compared to IP runners (119.30 ± 11.10 cm) with a large effect size (d = −0.84). Contact time does not significantly change (n-IP 0.30 ± 0.04, IP 0.30 ± 0.02, d = 0.06), and spm significantly varies between n-IP group (163 ± 13.90 spm) and IP group (170 ± 11.40 spm) with a medium effect size (d = −0.51). The vertical CoM displacement results were higher in the n-IP group (n-IP 6.20 ± 1.00 cm, IP 5.80 ± 1.40 cm) with a small effect size (d = 0.35). Furthermore, step cycle time, defined as number of steps in 1 s, did not considerably change (n-IP 1.43 ± 0.09 c/s, IP 1.46 ± 0.09 c/s, d = 0.33). Finally, running speed was not statistically different between the groups (n-IP 11.90 ± 1.50 km/h, IP 11.20 ± 2.90, d = −0.32).

### 3.2. Kinematic Parameters

Several differences were found between the impact peak and the no-impact peak groups. Three variables demonstrated a large effect size (d) greater than 0.80, while two proved a medium effect size (d) greater than 0.50. The means and effect sizes of the sagittal plane parameters are reported in Table 3. The trunk inclination did not statistically differ between the two groups. Hip flexion showed a statistically different range of motion in the n-IP group compared to the IP group (n-IP 32.30° ± 10.20, IP 40.40° ± 9.50) with a large effect size (d = −0.82). The hip extension shows a similar trend with a range of motion reduction in the n-IP group (n-IP 27.70° ± 4.60, IP 30.20° ± 3.90), reporting a medium effect size (d = −0.58). Knee flexion shows only a non-statistical difference with small effect sizes in both flexion and extension. The ankle dorsiflexion indicates a statistical difference between the groups. The n-IP group has a reduced range of motion (13.40° ± 7.20), compared to the IP group, which shows a completely rearfoot strike pattern (20.80° ± 5.50), with a large effect size (d = −1.17). Meanwhile, ankle plantarflexion between the groups shows a statistical difference however with a small effect size. Initial and final foot contact has been evaluated in the frontal plane. The foot inversion at initial contact statistically differs in the n-IP group compared to the IP group (n-IP 17.30° ± 3.80, IP 14.30° ± 3.50) with a large effect size (d = 0.83). A reduced foot inversion is present at the toe-off phase (n-IP 3.60° ± 3.00, IP 5.00° ± 2.70) with a medium effect size (d = −0.50).

### 3.3. Footwear and Injuries

Footwear differences were present among the runners, with a substantial difference in the HTD drop between IP and n-IP groups. The data are reported in Table 4. The HTD drop of the runners belonging to the IP group is 14.29% (8 mm), 42.86% (10 mm), and 42.86% (12 mm). Whereas the HTD drop of the runners belonging to the n-IP group is 57.14% (4 mm), 14.29% (6 mm), 14.29% (8 mm), and 14.29% (10 mm). Furthermore, we calculated the mean weight of the shoes; the IP group have a mean shoe weight of 273.43 ± 31.80 g, while the n-IP group have 244.0 ± 39.40 g. Concerning the injuries, 57.14% of n-IP runners did not experience an injury during the last year, while IP runners experienced an injury at least once (42.86%) or twice (42.80%) during the last year. Furthermore, the latter experienced a severe injury, and 50% of them reported the need for physical therapy to recover from the trauma.

### 3.4. Correlation Matrix

**Figure 1:**
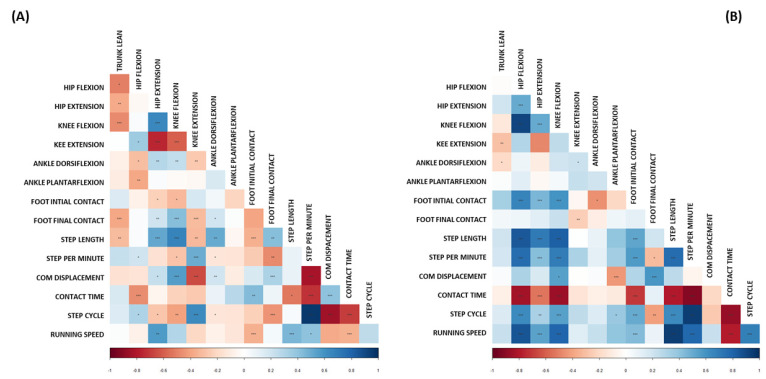
Correlation matrices of impact peak presence group (A) and impact peak absence group (**B**). The figure (**A**) presents a majority of negative correlations (red color); the figure (**B**) presents a majority of positive correlations (blue color). *** = *p* < 0.01, ** = *p* < 0.05, * = *p* < 0.1. The IP group demonstrated a general occurrence of negative correlations, according to matrix color. The n-IP group generally presented positive correlations.

Both groups underwent a Pearson correlation coefficients (r) analysis. The results are graphically shown in the correlation matrices, Figure 1. The IP group presented an overall incidence of negative correlations between the variables. Only moderate to strong correlations (r > ±0.50) with a *p* < 0.01 are discussed. Negatively correlated variables: hip extension with knee extension (r = −0.751); CoM vertical displacement with knee extension (r = −0.658); contact time with steps per minute (r = −0.729); CoM vertical displacement with steps per minute (r = −0.821); step time with CoM vertical displacement (r = −0.847); and contact time with step time (r = −0.699). Positively correlated variables: stride length with hip extension (r = 0.569); stride length with knee flexion (r = 0.724); stride length with foot dorsiflexion (r = 0.526); CoM vertical displacement with knee flexion (r = 0.550); and step time with knee extension (r = 0.630), Figure 1A.

Conversely, the n-IP group presented an overall incidence of strong positive correlations between the variables. Positively correlated variables: hip flexion with foot inversion at initial contact (r = 0.661); hip flexion with stride length (r = 0.842); hip flexion with steps per minute (r = 0.765); hip flexion with step time (r = 0.638); hip extension with stride length (r = 0.704); knee extension with foot inversion at initial contact (r = 0.596); knee flexion with stride length (r = 0.819); knee flexion with steps per minute (r = 0.691); foot inversion at initial contact with steps per minute (r = 0.567); foot inversion at toe-off with CoM vertical displacement (r = 0.581); stride length with steps per minute (r = 0.761); running speed with hip flexion (r = 0.85), hip extension (r = 0.533), knee flexion (r = 0.794), spm (r = 0.785), and step cycle (r = 0.684). While, the only variable showing strong negative correlations for n-IP group was contact time with: hip flexion (r = −0.839), knee flexion (r = −0.852), foot inversion at initial contact (r = −0.701), stride length (r = −0.798), steps per minute (r = −0.919), and running speed (r = −0.788), as shown in Figure 1B.

## 4. Discussion

**Figure 2:**
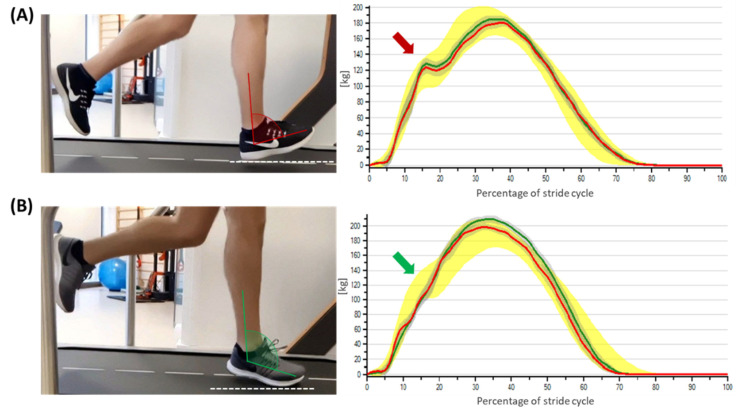
The impact peak force graphs showing impact peak presence (**A**), the red arrow specifies the impact peak occurrence; and impact peak absence (**B**), the green arrow specifies the impact peak absence. The red line represents the left side of the body, the green line represents the right side, the yellow band represents normal values.

This study aimed to analyze the running pattern of recreational runners through a markerless system and to determine whether it has a connection with the presence/absence of an impact peak. We measured the running patterns according to differences in the 3D gait kinematics of the hip, knee, ankle, foot, and spatiotemporal parameters. The sample classification for the presence or absence of the impact peak adequately matched the runners according to the main biomechanical joint characteristics of rearfoot (Figure 2A) and forefoot runners (Figure 2B) [3]. The results suggest that recreational runners without the impact peak present a shorter stride length, reduced hip flexion, increased foot inversion at initial contact, and predominantly reduced ankle dorsiflexion at initial contact compared with the group with the absence of the impact peak.

In our sample, runners that exhibited a reduction in stride length did not present an impact peak. Various authors [27,28] agree that shock attenuation changes only when stride length changes. We support that it is plausible that the association between an impact peak and stride length may vary due to leg geometry changes as stride length changes [27]. Differences in step frequency are present between the groups. The n-IP group has a lower step frequency (163 ± 13.90 spm) than the IP group (170 ± 11.40 spm). It has been shown to be lower in several studies [4,29,30], furthermore the impact peak absence is correlated to an SPM frequency of 180 spm. This could be affected by the running speed of the study sample because it was self-selected by the runners, and it was probably not their maximal speed. Secondly, there is also a positive correlation between step frequency and hip flexion, hip extension, and knee flexion. Since all values were lower in the n-IP group, we hypothesize that this reduction could be related to the lower step frequency. Even when the CoM vertical displacement had a small effect size, there was still a noticeable difference, whereas the n-IP group tended to present a higher CoM displacement, although generally, it was lower that reported for recreational runners from two previous studies [31,32]. Furthermore, Shih et al. [31] stated that the vertical displacement of CoM does not statistically differ among barefoot and shod runners or rearfoot and forefoot strikers. Our sample showed a reduced vertical displacement. The increased hip extension may explain a CoM drop during the single support phase, which determines a reduced CoM vertical displacement. Secondly, the use of the treadmill could be a reason for a reduced CoM displacement, compared tp alternative running environments that can add moderate effects to the vertical displacement [33].

The study findings showed no difference in the trunk forward lean between n-IP and IP groups. This phenomenon may have occurred because participants ran at a self-paced speed, and when this condition is met, runners are not prone to increase their trunk inclination [34]. Weinhandl et al. [35] and Sah et al. [36] described the trunk flexion increase as a compensatory strategy to modulate shock attenuation during the run, while Hart et al. [37] showed that paraspinal muscular fatigue could increase trunk flexion. In contrast to these findings, there was no difference in trunk inclination between the n-IP and IP groups. The runners had not trained before the data acquisition, so fatigue was not an important factor [38,39]. The WalkerView can easily detect trunk inclination, and therefore, it can easily educate recreational runners to increase trunk flexion and reduce patellofemoral joint stress, as the literature suggests [34,35].

Our results show a reduction in hip flexion and an increase in hip extension in the n-IP group, compared to other studies [40,41]. Hip flexion data differs from Dos Santos et al. [42], that reported that the hip flexion appears to increase from rearfoot to forefoot runners. Even if the strike pattern was not categorized our sample, the n-IP group’s ankle dorsiflexion corresponds to midfoot/forefoot runners. Consequently, a hip flexion reduction was demonstrated in those without an impact peak [6]. Knee flexion was similar to the findings of Koblbauer et al. findings [41], in contrast to the findings of Rueda et al. study [40], where it appears reduced. A possible explanation may be that reduced hip flexion limits knee motion and recreational runners’ tend to reach the surface with less knee flexion due to foot placement being further away from the center of mass [40].

Wang et al. [29] evaluated the changes in lower extremity biomechanics in recreational runners after a 12-week training protocol. By comparing the ankle angle at initial contact, our IP group is comparable to this study’s pre-training group, while the post-training group is similar to our n-IP ankle group angle at initial contact. That value lies within the range of 8°–15°, reported as the correct ankle range at initial contact to prevent peak force impact [43]. Our n-IP group did not show a real forefoot strike pattern; nevertheless, adopting a midfoot strike pattern can reduce the load rate by around 50% and perhaps altogether remove the impact peak [4,44,45]. Moen et al. [46] highlighted that reducing excessive ankle dorsiflexion can increase the stress on the shank muscles and joints. However, as our sample group has more than ten years of running experience, this should not be a significant issue. This precaution must be carefully considered in those who intend to change their strike pattern.

Our results indicate that runners overcoming the impact peak presence exhibit a reduced hip range of motion for flexion and extension. A slight increase of knee flexion potentially supports a hip flexion reduction. The findings on ankle dorsiflexion at initial contact align with all the previous studies investigating this particular joint as the leading factor of impact peak reduction [3]. An increase in foot inversion at initial contact was also present. Both recreational runners and trainers should be aware of excessive hip flexion, ankle dorsiflexion, stride length, and reduced foot inversion at initial contact because these factors may predispose runners to running-related injuries. However, running-related injuries are not directly correlated with foot strike patterns. Burke et al. [47] recently highlighted that there is little evidence to suggest a relationship between these two conditions. Accordingly, we observed the impact peak predominantly in a sample of rearfoot runners; however, we do not speculate about the relationship between the strike pattern and the injury onset.

Secondly, we surveyed the runners about their footwear to identify the main characteristics according to the classification of IP and n-IP groups. The IP group generally wears shoes with a high HTD and they are used to buying shoes that are half a size(EU sizes) greater than the usual size of their non-sports shoes. Meanwhile, the n-IP generally wear shoes with a low HTD and almost all of them are used to buying the shoes one size greater than the usual size, which may explain why 85.71% of them do not experience pain in their feet at the end of training. Regarding the incidence of injuries, the IP group had a greater proportion of runners that experienced an injury during the last year, with the knee as the location with the higher incidence. The HTD should not influence the injury risk, actually, low HTD could be associated with higher injury risk in regular runners [48]. The information collected about the injury location is similar to Kakouris et al. [49] who found the knee as the location with the highest rate of injury for the IP group. Meanwhile, the n-IP group did not align with the previous trend, having hamstrings and calf muscles as the zones with the highest proportion. However, we cannot unequivocally correlate the use of different footwear with the incidence of the impact peak because we observed the behavior of recreational runners concerning the use of the shoes and their aspects rather than analyzing different shoes in the biomechanics analysis.

This study’s results have potential scientific relevance for training programs for runners with an impact peak. When a recreational runner’s hip extension is around 40°, ankle dorsiflexion is around 20°, and initial foot contact is around 14°, they may fit the profile of a runner with an impact peak. Furthermore, a stride length exceeding 120 cm can negatively affect the performance of recreational runners.

Certain limitations should be considered, however, when interpreting these findings. First, we analyzed only sagittal kinematics through a 3D markerless system, whose accuracy cannot be compared with marker-based systems. Secondly, the analysis took place on a treadmill, so results have to be considered carefully compared to overground running.

Further studies should include a more significant number of participants, ideally in an outdoor setting, and investigate the differences in the running pattern with their own shoes compared with standard shoes. Further attention is required as recreational runners are highly likely to be injured [50]. Many studies have examined only specific running characteristics [51], omitting any possible interaction between spatiotemporal, kinetic, kinematic parameters, and footwear. Awareness of the specific biomechanical factors behind the onset of pain or injury can help clinicians select a well-suited treatment strategy. However, it is challenging to manage appropriate injury prevention programs without further studies regarding the biomechanical factors that precede an injury [52].

## 5. Conclusions

Recreational runners without an impact peak commonly run with low HTD drop shoes, exhibit a shorter stride length, and demonstrate a slight increase in CoM vertical displacement. Trunk forward lean does not differ between the groups. Hip flexion is reduced, balancing the ankle dorsiflexion and foot inversion at initial contact. Finally, this study emphasizes an approach based on a 3D motion capture markerless system analysis, which may easily and quickly elucidate the complex correlations of the impact peak presence. Sports physicians and coaches are called upon to collect more information about running-related injuries in recreational runners to prevent possible chronic disabling diseases. Furthermore, it is essential to design training programs with a well-suited approach because recreational runners are more numerous than professionals. In the future, we aspire to contribute new information to the scientific community about running through 3D analysis to help prevent injury and enhance the performance of recreational runners.

## Figures and Tables

**Table 1:** Participants information.

M	Mean Angle (SD)	
	IP group	n-IP group
Age (years)	47.19 (6.85)	45.15 (6.09)
Height (cm)	174.38 (5.10)	174.85 (6.91)
Body mass (kg)	70.81 (6.06)	73.15 (7.60)
BMI (kg/m^2^)	23.27 (1.45)	23.92 (1.94)
Weekly km (km)	40.00 (6.32)	44.20 (14.97)
Weekly training (days)	3.34 (0.52)	3.50 (1.05)

IP group = impact peak presence; n-IP = impact peak absence; BMI = body mass index.

**Table 2:** Spatiotemporal parameters of recreational runners.

	Mean Angle (SD)				
	IP Group	n-IP Group	Sig.	Effect Size (d) ^+^	U3 ^++^
Stride length (cm)	105.50 (20.50)	119.30 (11.10)	0.02 *	**−0.84**	*80%*
Contact time (s)	0.30 (0.04)	0.30 (0.02)	0.40	0.32	-
SPM	163 (13.90)	170 (11.40)	0.17	***−0.51***	*69.5%*
CoM displacement (cm)	6.20 (0.98)	5.80 (1.39)	0.37	0.35	*-*
Step cycle (c/s)	1.43 (0.09)	1.46 (0.09)	0.46	−0.33	*-*
Speed (km/h)	11.20 (2.88)	11.90 (1.50)	0.18	−0.32	*-*

IP group = impact peak presence; n-IP = impact peak absence; **^+^** Cohen’s values; ^++^ Cohen’s U3 describes the proportion of distribution overlap; Sig. according to *t*-test for normal data and Mann–Whitney U for non-normal data (* < 0.05). Note: bold numbers indicate a large effect size between groups (d > 0.80). Bold and italic numbers indicate a medium effect size between groups (d > 0.50).

**Table 3:** Kinematic parameters of recreational runners.

Joint Excursion	Mean Angle (SD)				
	IP Group	n-IP Group	Sig.	Effect Size (d) ^+^	U3 ^++^
*Trunk*					
Flexion	11.40° (2.30)	11.60° (2.40)	0.31	−0.06	-
*Hip*					
Flexion	40.40° (9.50)	32.30° (10.20)	0.03 *	**−0.82**	*79.3%*
Extension	30.20° (3.90)	27.70° (4.60)	0.12	***−0.58***	*71.9%*
*Knee*					
Flexion	86.60° (17.10)	88.74° (15.80)	0.73	0.13	*-*
Extension	5.60° (3.50)	4.90° (3.40)	0.59	−0.20	*-*
*Ankle*					
Dorsiflexion	20.80° (5.50)	13.40° (7.20)	0.003 **	**−1.17**	*87.9%*
Plantarflexion	50.30° (4.60)	51.50° (4.70)	0.03 *	0.25	*-*
*Foot*					
Inversion at IC	14.30° (3.50)	17.30° (3.80)	0.03 *	**0.83**	79.6%
Inversion at TO	5.00° (2.70)	3.58° (3.00)	0.13	***−0.50***	*69.2%*

IP group = impact peak presence; n-IP = impact peak absence; **^+^** Cohen’s values; ^++^ Cohen’s U3 describes the proportion of distribution overlap; Sig. according to *t*-test for normal data and Mann–Whitney U for non-normal data (* < 0.05, ** < 0.01); IC = initial contact; TO = toe-off. Note: bold numbers indicate a large effect size between groups (d > 0.80). Bold and italic numbers indicate a medium effect size between groups (d > 0.50).

**Table 4:** Specific conditions related to footwear and the incidence of injuries.

	n-IP Group	IP Group
*Shoes size*		
Same as the foot size	28.57%	14.29%
½ point greater	14.29%	57.14%
1 point greater	57.14%	28.57%
*New shoes change*		
After 600–800 km	71.43%	71.43%
After 800–1000 km	28.57%	-
When the shoes are ruined	-	28.58%
*Feet pain after training*		
Yes	14.29%	57.14%
No	85.71%	42.86%
*Suspend due to injury (in one year)*		
0 times	57.14%	14.29%
1 time	28.57%	42.86%
2 times	14.29%	42.80%
*Common injury location*		
Back	7.14%	-
Hip	7.14%	-
Hamstring	21.43%	16.67%
Knee	7.14%	41.67%
Calf	21.43%	16.67%
Achilles tendon	-	8.33%
Foot	-	8.49%
None	35.72%	8.33%
*Injury severity*		
None	42.86%	12.50%
Mild, needed a little rest	28.57%	25.00%
Moderate, extended rest and ice	28.57%	12.50%
Severe, needed medications or physiotherapy	-	50.00%

## Data Availability

The data of this study are available upon request.

## Supplementary Materials

The following supporting information can be downloaded at: https://www.mdpi.com/article/10.3390/biology11060818/s1, Supplementary File S1: Recreational runners behavior questionnaire.
