# Supplementary material for: Running Footwear and Impact Peak Differences in Recreational Runners
Source: Biology (Basel). 2022 May 26;11(6):818. doi: 10.3390/biology11060818 (PMC9219650; doi:10.3390/biology11060818)
Supplement: Supplementary file 1 [file biology-11-00818-s001.zip › biology-1731441-supplementary.pdf]

Supplementary File S1: RECREATIONAL RUNNERS BEHAVIOR QUESTIONNAIRE

What kind of shoes do you use for running? (write the brand, type. e.g: Nike Vomero 16)

Answer___________________

The shoes are the same number size of your feet?
- Yes
- No, I buy them half point larger
- No, I buy them always 1 point larger
- No, I buy them always 2 points larger

Do you use only this pair of shoes to run?
- Yes
- No, I switch them with a different pair when these hurts
- No, I switch them but only with the same model of shoes
- I do not pay attention to the shoes that I use to run

How often do you change your running shoes?
- Every 600-800 km
- Every 800-1000 km
- When my feet start hurt
- When the sole of the shoes starts to get ruined

Do you use any particular sole? (es: Noene, shock absorbing, ecc)
- No
- Yes (if yes, specify the type)
- Other

Do your feet hurt when you sto running?
- Yes
- No
- Sometimes, but non every time

How much kilometers do you run per week?

Answer____________

How many times do you run on average per week?
- 1
- 2
- 3
- 4
- 5
- 6

How many times in the last year did you have to stop for at least two weeks due to an injury?

- 0
- 1
- 2
- 3
- 4
- 5

Choose which region of interest had traumas in the last year?

- Hip
- Groin
- Posterior thigh
- Anterior thigh
- Knee
- Shin
- Calf
- Foot
- Achilles tendon
- None of these

If you had a trauma, it was:

- Mild, I just had to rest and it went away alone
- Moderate, I needed prolonged rest and ice
- Severe, I had to take drugs or undergo physiotherapy

Do you practice any other sport aside from running?

- No
- Yes (if yes, specify which)
- Other
